# Resource Use and Cost Comparison of Mohs Micrographic Surgery vs Traditional Surgery for High-risk Facial Basal Cell Carcinoma

**DOI:** 10.2340/actadv.v106.adv-2025-0263

**Published:** 2026-07-09

**Authors:** Julia Fougelberg, Lovisa Klein, Emelie Pauli, Ann-Marie Wennberg Larkö, Hannah Ceder

**Affiliations:** 1 Department of Dermatology and Venereology, Institute of Clinical Sciences, Sahlgrenska Academy, University of Gothenburg, Gothenburg, Sweden; 2 Region Västra Götaland, Sahlgrenska University Hospital, Department of Dermatology and Venereology, Gothenburg, Sweden; 3 Region Västra Götaland, Sahlgrenska University Hospital, Gothia Forum for Clinical Trials, Gothenburg, Sweden

**Keywords:** Mohs micrographic surgery, keratinocyte cancer, non-melanoma skin cancer, basal cell carcinoma, health resources, cost-benefit analysis

## Abstract

High-risk facial basal cell carcinomas are preferably treated with Mohs micrographic surgery, but despite its documented benefits, it remains underused in Sweden. The aim of this study was to evaluate healthcare resource utilization and direct costs for patients with high-risk facial basal cell carci-nomas treated with Mohs micrographic surgery compared with traditional surgical excision. In this retrospective registry study, all patients undergoing Mohs micrographic surgery or surgical excision at Sahlgrenska University Hospital between 2020 and 2024 were included, supplemented by regional healthcare data from the Vega registry, to assess long-term healthcare use up to 10 years after primary treatment. Mohs micrographic surgery was associated with higher initial costs than surgical excision but a slower accumulation of costs over time. Patients initially treated with surgical excision who later required Mohs micrographic surgery incurred the highest total costs, often after multiple prior procedures. These findings indicate that although Mohs micrographic surgery is costlier initially, early access prevents the substantial downstream costs associated with treatment failure. Primary Mohs micrographic surgery is a cost-efficient strategy for high-risk facial basal cell carcinomas and should be used more frequently in order to enable better utilization of resources and improved outcomes for the patient.

**SIGNIFICANCE**

Mohs micrographic surgery for high-risk facial basal cell carcinomas is underused in the Nordic countries, probably due to limited access but also due to higher resource utilization and costs. This study demonstrates that Mohs micrographic surgery, despite its higher initial cost, reduces the need for subsequent interventions and ongoing healthcare utilization. These findings clarify the long-term economic and clinical value of selecting Mohs surgery at an early stage. By providing evidence for more efficient allocation of healthcare resources, the study contributes to improved treatment strategies and supports policies that enhance patient outcomes while reducing overall societal costs.

Basal cell carcinoma (BCC) represents a growing public health concern in fair-skinned populations, with over 71,000 new cases reported in Sweden in 2023 (1, 2). The rising incidence, particularly among high-risk subtypes underscores the need for cost-effective treatments that reduce morbidity and patient suffering (3). High-risk facial BCCs are preferably treated with Mohs micrographic surgery (MMS), which offers superior cure rates and tissue-sparing benefits (4). While MMS is widely practiced in the USA and parts of Europe, its use remains limited in Scandinavia (5–8).

In Sweden, all BCC tumours are classified by histopathological subtype according to the Sabbatsberg-model. This system categorizes tumours into 3 risk groups: (*i*) “low risk” (Glas type IA and IB), (*ii*) “medium risk” (Glas type II) and (*iii*) “high risk” (Glas type III). Type II includes less aggressive infiltrative or micronodular BCCs, with clearly demarcated borders and invasion depth limited to the dermis. Type III can have infiltrative, micronodular, morpheaform or basosquamous growth patterns, with perineural and/or perivascular growth. The tumour borders are irregular without any clear demarcation, with tumour nests arranged in small (1–2 cells thick) irregular cords of basaloid cells, extending into the dermis or even subcutis, muscle, cartilage and/or bone (9). In this paper, type II and III BCCs (according to the Swedish classification) are referred to as high-risk subtypes due to their shared features.

There are many challenges in the management of high-risk facial BCCs. These include diagnostic difficulties across medical specialties, limited access to MMS, maybe because of insufficient knowledge of its indications and benefits but also long waiting times (up to 1 year in Sweden). Unreliable preoperative biopsies that can misguide clinical decision-making are also a challenge (10–12). Together, these factors contribute to suboptimal treatments, leading to high rates of incomplete excisions (8, 13–15) and recurrences (16–20). Traditional surgery (surgical excision, SE) for high-risk subtypes requires wider margins (5–15 mm) (4, 7, 21–23) and several studies report that 25–55% of high-risk facial BCCs are incompletely excised with SE (8, 13–15). The conventional technique for histopathological examination (“bread-loafing”) has limited coverage, only examining 0.1–2% of the surgical margin, compared to MMS’s 100% margin assessment (5, 16, 20, 21, 24, 25). Thus, MMS allows for effective detection of subclinical extensions (i.e. tumour growth invisible to the naked eye during clinical examination), while saving maximum healthy tissue (26).

Although MMS is not a cutting-edge method (27), its rarely performed in Scandinavia due to limited resources (5, 16). In Sweden, MMS is reserved for high-risk facial BCCs (28). Despite this, only about 5% of eligible patients receive MMS. Currently, the capacity is confined to three centres (Stockholm, Gothenburg and Lund), which together perform approximately 600 procedures annually. Given that at least 11,000 high-risk BCCs are diagnosed per year (3), there is a substantial gap between demand and MMS capacity. This gap might be due to this limit of access but probably also due to higher resource utilization and costs. Data on the cost-effectiveness of MMS vs SE are limited, with no consensus on which surgical method offers better value for the money. To the best of our knowledge, no such study has been conducted in Scandinavia.

## Objectives

The aim of this study was to explore the health economic potential of MMS as a treatment for high-risk facial BCCs. The overall objective was to compare health care resource utilization for patients who received primary MMS and those who primary underwent traditional surgery or other treatments. Also, we wanted to analyse potential differences in terms of gender, age, comorbidity and geographical residence for those who underwent different primary treatments.

## MATERIALS AND METHODS

This retrospective registry study was conducted at the Department of Dermatology at Sahlgrenska University Hospital, Gothenburg, Sweden. Consecutive patients treated with MMS or SE for high-risk facial BCC (type II and III according to the Swedish classification) between 2020 and 2024 were included. The study was approved by The Swedish Ethical Review Authority.

Healthcare utilization data were obtained from the Vega registry, Region Västra Götaland’s database for healthcare resource use. Diagnoses were identified using International Classification of Diseases (ICD-10) codes and procedures using the Swedish Classification of Health Interventions (KVÅ codes).

The study population comprised 2 cohorts: (*i*) patients registered in the local MMS registry at our department and (*ii*) patients identified in the Vega registry with high-risk facial BCC (ICD-10: C443C, C443D, C443E) referred for MMS or treated with SE or other destructive treatments (e.g. cryosurgery, curettage and electrodessication) (Table I). Data from 2014 to 2024 enabled retrospective assessment of treatment pathways before MMS vs MMS as primary treatment.

**Table I.** ICD-10 codes used to define cohort 2

ICD-10 code	Description
C441-C	Malignant neoplasm of skin of lip (excl. vermilion border)
C441-D	Malignant neoplasm of skin of external ear
C442-D	Malignant neoplasm of skin of eyelid, including canthus
C443-C	Malignant neoplasm of skin of nose
C443-D	Malignant neoplasm of skin of scalp and neck
C443-E	Malignant neoplasm of skin of other and unspecified parts of face

The following variables were collected; age, sex, date of diagnosis, date of surgery, tumour location, histopathological subtype, healthcare utilization and cost per patient (CPP). Patients were categorized into MMS, SE or destructive treatment groups based on KVÅ codes and ICD-10 codes (Table II). Inclusion was restricted to patients considered potentially eligible for MMS.

**Table II.** Classification of treatment based on KVÅ codes

Treatment	KVÅ codes	Description
Surgical excision (SE)	QAE10 without ZQX00	Surgical excision in the head and neck region
Mohs micrographic surgery (MMS)	QAE10 in combination with ZQX00	Surgical excision in the head and neck region combined with micrographic technique (Mohs surgery)
Other treatments	CAW99	Other orbital surgery
	CBB30	Excision of tumour or local lesion in eyelid
	CBB32	Destruction of local lesion in eyelid
	CBB50	Excision of tumour/lesion in eyelid with reconstruction using graft or flap
	CBB99	Other removal of tumour/lesion in eyelid
	CBD10	Reconstruction of eyelid with graft or flap
	CBD11	Opening of flap or eyelid reconstruction
	DHB00	Excision from external nose, e.g., tumour
	DHW99	Other nasal surgery
	DQ004	Photodynamic therapy (PDT)
	DV070	Radiotherapy
	QAA25	Destruction of skin lesion in head and neck region
	QAE00	Excision in head and neck region
	QXA25	Destruction of skin lesion, unspecified region
	ZXC00	Use of diathermy
	ZXC50	Use of cryotherapy
	ZXC65	Curettage
	ZXH20	Nerve block anaesthesia
	ZZA50	Free full-thickness skin graft

### Statistical analysis

Data were analysed using Stata version 17.0 (StataCorp, College Station, Texas, USA) in collaboration with Gothia Forum health economists. Missing CPP values were estimated using Diagnosis Related Group (DRG) weights and the 2024 regional base tariff. Costs were adjusted to 2024 SEK using the Swedish Consumer Price Index and converted to EUR using the average exchange rate for 2024 (1EUR=11.43) from the Swedish Central Bank (Riksbanken).

Costs were analysed at 1, 3, 5 and 10 years after primary treatment. To address baseline differences between treatment groups, multivariable linear regression analyses were performed adjusting for age, sex, comorbidity burden and municipal category.

## RESULTS

### Demographics

**Fig. 1.**
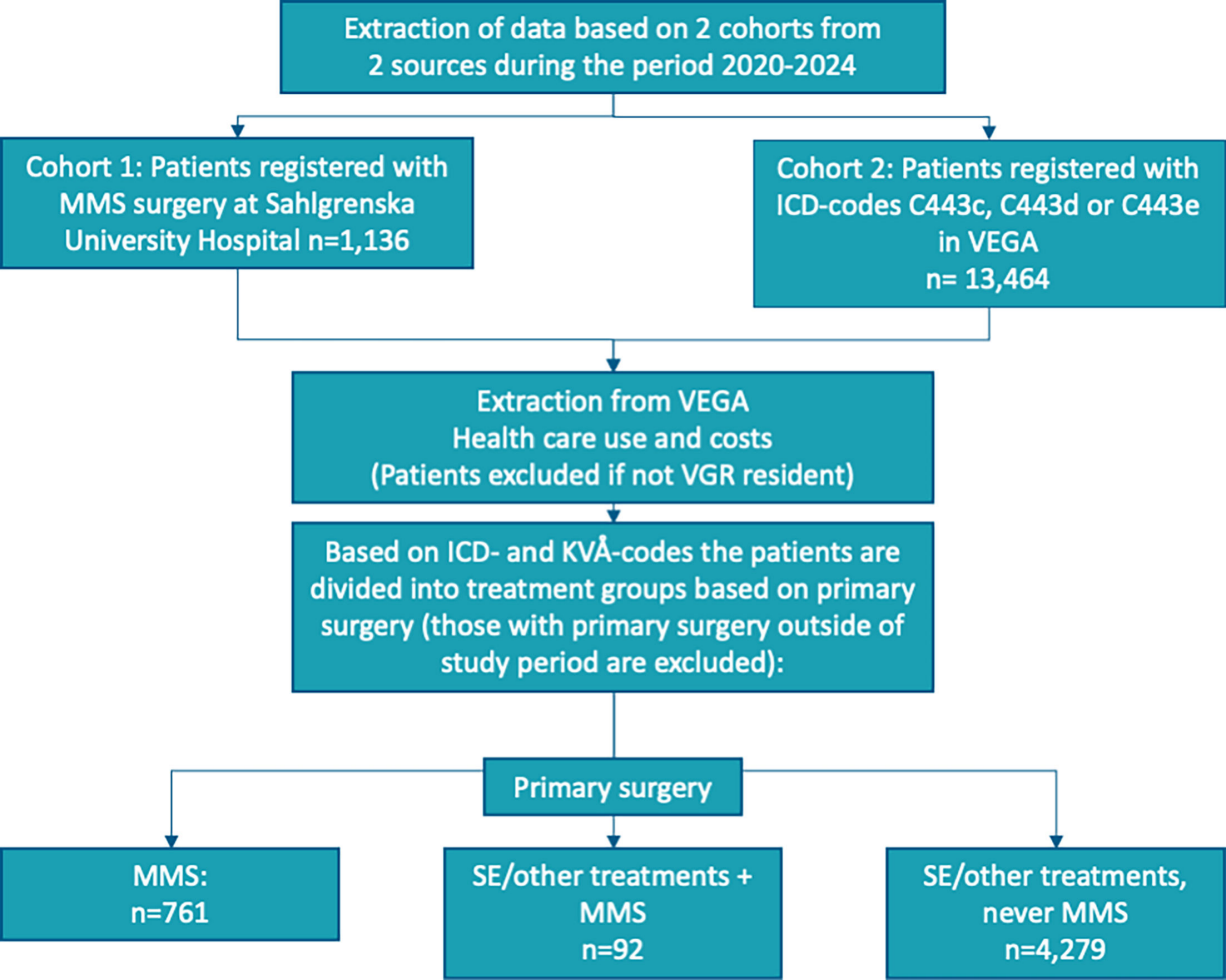
Flow chart of extraction process.

Among 5,132 patients with high-risk facial BCC, 2% received initial SE followed by MMS (*n*=92), 83% were treated with SE without MMS (*n*=4,279), and 15% underwent primary MMS (*n*=761) (Fig. 1).

Patients in the SE-to-MMS group were slightly younger (median age of 71 years, IQR 63–77), compared with the SE-only group (76 years, IQR 68–82) and the primary MMS group (72 years, IQR 61–78) (Table III). The SE-only group had a higher proportion of patients ≥80 years (34%), whereas the MMS group had a greater proportion of patients aged 50–69 years.

**Table III.** Demographics

Patient group	SE/other, then MMS *n*=91			SE/other, never MMS *n*=4,282		Primary MMS *n*=766	
Age, years, median (IQR), range	71 (63–77, 42–89)			76 (68–82, 23–105)		72 (61–78, 17–92)	
Age categories	*n*	%		*n*	%	*n*	%
20–29 y	−	−		5.0	0.1	2.0	0.3
30–39 y	−	−		32.0	0.8	20.0	2.6
40–49 y	9.0	9.9		143.0	3.3	49.0	6.4
50–59 y	7.0	7.7		355.0	8.3	106.0	13.9
60–69 y	24.0	26.4		719.0	16.8	156.0	20.5
70–79 y	38.0	41.8		1,560.0	36.5	286.0	37.5
80–89 y	13.0	14.3		1,233.0	28.8	137.0	18.0
90–99 y	−	−		229.0	5.4	6.0	0.8
≥100y	−	−		4.0	0.1	−	−
Sex							
Male	38.0	41.8		2,023.0	47.3	302.0	39.6
Female	53.0	58.2		2,256.0	52.7	461.0	60.4
Home municipality*							
Group A: large cities/close to large city	74.0	81.3		831.0	37.7	594.0	77.9
Group B: medium-sized towns	14.0	15.4		911.0	41.3	84.0	11.0
Group C: smaller towns/rural	3.0	3.3		464.0	21.0	85.0	11.1
Comorbidities - ICD-10-chapters**							
A: Certain infectious and parasitic diseases	−	−		−	−	−	−
B: Certain infectious and parasitic diseases (cont.)	−	−		2.0	0.1	−	−
C: Neoplasms (high-risk BCC excluded)	5.0	5.3		372.0	8.2	19.0	2.4
D: Diseases of the blood and blood-forming organs	2.0	2.2		277.0	6.2	21.0	2.7
E: Endocrine, nutritional and metabolic diseases	2.0	2.2		51.0	1.2	2.0	0.3
F: Mental and behavioural disorders	−	−		19.0	0.4	−	−
G: Diseases of the nervous system	−	−		17.0	0.4	−	−
H: Diseases of the eye, ear, etc.	−	−		15.0	0.4	−	−
I: Diseases of the circulatory system	5.0	5.2		114.0	2.6	5.0	0.7
J: Diseases of the respiratory system	−	−		21.0	0.5	2.0	0.3
K: Diseases of the digestive system	−	−		4.0	0.1	−	−
L: Diseases of the skin and subcutaneous tissue	7.0	7.2		631.0	13.4	22.0	2.8
M: Diseases of the musculoskeletal system	−	−		13.0	0.3	1.0	0.1
N: Diseases of the genitourinary system	1.0	1.1		13.0	0.3	−	−
O: Pregnancy, childbirth and the puerperium	−	−		−	−	−	−
Q: Congenital malformations, deformations and chromosomal abnormalities	−	−		3.0	0.1	−	−
R: Symptoms, signs and abnormal clinical findings	1.0	1.1		19.0	0.4	2.0	0.3
Z: Factors influencing health status	6.0	6.1		148.0	3.4	15.0	1.9

*Based on SALAR’s definition from 2023: A. Large cities and municipalities near large cities, B. Medium-sized towns and municipalities near medium-sized towns, C. Smaller towns/urban areas and rural municipalities.

**Based on other diagnoses registered at time of primary treatment.

Women comprised 58% of the SE-to-MMS group, 53% of the SE-only group, and 60% of the primary MMS group. Both MMS groups were more frequently urban residents (81% and 78% respectively), compared with 38% in the SE-only group.

Co-morbidity was generally lower among MMS-treated patients. The SE-only group had the highest prevalence of comorbid conditions, particularly skin diseases (13%), neoplasms (8%) and blood disorders (6%), whereas primary MMS patients had the lowest overall comorbidity rates. Overall, MMS-treated were younger, more often urban, and had fewer comorbidities than SE-only patients.

### Resource use and costs

Patients undergoing SE prior to MMS represented the most resource-intensive subgroup, with a mean of 3 prior SE procedures (Table IV).

**Table IV.** Total treatment costs in SEK for patients undergoing one to six failed SEs before MMS

Number of SEs	Cost for SEs (€)	Cost for MMS (€)	Total treatment cost
1	685	1,889	2,574
2	1,370	1,889	3,256
3	2,055	1,889	3,944
4	2,740	1,889	4,629
5	3,425	1,889	5,314
6	4,110	1,889	5,999

Table V presents cumulative costs up to 10 years. In the first 10 year, costs were highest for primary MMS (€ 1,889) and lowest for SE-only treatment (€ 685). Over time, costs increased in all groups, but most markedly among patients who ultimately required MMS after SE, reaching € 3,853 at 10 years. The SE-only group showed a gradual increase from € 685 to € 1,892, while primary MMS costs rose more moderately to € 2,856 at 10 years. Specialist outpatient care accounted for the majority of total costs across all groups, whereas inpatient and primary care costs remained low.

**Table V.** Mean direct healthcare cost for high-risk BCC related healthcare per person in SEK, mean and 95% CI

	**Cost per patient**								
**Primary surgery**	**SE/other, then MMS**			**SE/other, never MMS**			**MMS**		
**Up to 1 year following primary surgery**	***n*=92**			***n*=4,279**			***n*=761**		
	**EUR**	**95% CI**		**EUR**	**95% CI**		**EUR**	**95% CI**	
Primary care	25	4	46	38	35	42	48	34	61
Outpatient spec	798	636	961	615	598	632	1,841	1,808	2,137
Inpatient	–			32	13	50	–		
Mean total per patient	823	660	986	685	658	712	1,889	1,853	1,925
**Up to 3 years following primary surgery**	**n=35**			**n=719**			**n=68**		
*Primary care*	67	10	124	108	91	125	138	59	216
*Outpatient spec*	1,094	811	1,377	869	808	931	2,352	2,085	2,619
*Inpatient*	–			92	33	151	–		
Mean total per patient	1,161	845	1,477	1,069	966	1,173	2,490	2,216	2,764
**Up to 5 years following primary surgery**	***n*=23**			***n*=418**			***n*=34**		
*Primary care*	139	38	241	129	106	152	109	-15	232
*Outpatient spec*	1,315	884	1,747	1,005	802	1,208	2,445	2,048	2,843
*Inpatient*	–			52	0	104	–		
Mean total per patient	1,454	953	1,956	1,186	933	1,438	2,554	3,001	2,981
**Up to 10 years following primary surgery**	***n*=12**			***n*=220**			***n*=12**		
*Primary care*	231	52	410	181	136	227	62	3	121
*Outpatient spec*	1,733	939	2 527	1,498	1,105	1,890	2,794	1,851	3,736
*Inpatient*	–			213	-2	429	–		
Mean total per patient	1,964	1,054	2,874	1,892	1,364	2,420	2,856	1,910	3,802
Mean total per patient (including MMS for group 1)	3,853								

Confidence intervals widened with longer follow-up, particularly in the MMS group, reflecting smaller sample sizes. In adjusted analyses (Table VI), primary MMS was associated with higher costs at 1 and 3 years compared with SE, but differences were attenuated and no longer significant at 5 and 10 years. Adjustment for age and comorbidity slightly influenced estimates, whereas sex and municipal category were not significant predictors.

**Table VI.** Multivariable regression analysis adjusting for age, sex, comorbidity and municipal category

Time horizon	SE/other never MMS	Primary MMS	Age	Female	Comorbidity index	R²	*N*
1 year	−186* (−364 to −8)	1,036*** (854 to 1,217)	1.47 (*p*=0.058)	NS	72* (6 to 139)	0.20	5,128
3 years	−251 (NS)	1,060*** (609 to 1,510)	8.47**	NS	NS	0.07	819
5 years	−600* (−1,174 to −27)	576 (*p*=0.086)	NS	NS	NS	0.03	474
10 years	NS	NS	NS	NS	(trend) *p*=0.078	0.03	244

Coefficients represent adjusted mean cost differences (EUR).

Robust standard errors used.

*p<0.05, **p<0.01, ***p<0.001.

NS: not statistically significant.

## DISCUSSION

Our findings indicate that the choice of surgical treatment pathway for high-risk facial BCCs has important implications for healthcare costs and resource use. Primary MMS was associated with higher initial healthcare costs (€ 1,889), consistent with its more resource-intensive nature. However, cost accumulation over time was slower compared with SE-based strategies, suggesting reduced need for subsequent interventions.

In contrast, SE or other procedures had lower upfront costs (€ 685) but showed a gradual increase, reaching € 1,892 at 10 years. The SE-to-MMS group had the highest cumulative costs (€ 3,853), reflecting repeated procedures and subsequent MMS. These findings highlight that treatment pathways with low initial costs may result in higher long-term expenditure when re-interventions are required.

Adjusted analyses indicated that differences in age, comorbidity and municipal category did not materially explain cost differences between treatment groups. However, long-term estimates should be interpreted with caution due to smaller sample sizes and wide confidence intervals.

The economic value of MMS depends on the proportion of patients requiring additional surgery after initial SE. In our cohort, 17% underwent MMS (15% primary, 2% after SE). Data from our Mohs registry show that at least 30% of the patients received other treatments before MMS (8). Previous studies have reported incomplete excision rates of 25–55% for high-risk facial BCCs, suggesting that the true proportion requiring MMS may be higher.

The most costly pathway was SE followed by MMS, while successful primary SE was the least costly. This supports that treatment failure has substantial economic consequences. However, our estimate of a “break-even” threshold (>50% MMS requirement) should be interpreted cautiously, as it is based on simplified cost assumptions and direct costs only. Indirect costs such as travel time, productivity loss and psychosocial burden were not captured. Similarly, clinical outcomes such as recurrence and re-interventions were not included. These limitations are inherent to registry-based cost analyses and likely underestimate the relative value of MMS.

Previous literature shows conflicting results regarding the cost-effectiveness of MMS vs SE, reflecting differences in healthcare systems, follow-up structures and study design (29–32). Most studies originate from insurance-based systems, limiting comparability with Nordic healthcare settings.

Patients treated with MMS were younger, more often female, and more urban, while SE-only patients were older and had higher comorbidity burden. Although these factors influenced short-term costs, they did not significantly alter between-group cost differences.

**Fig. 2.**
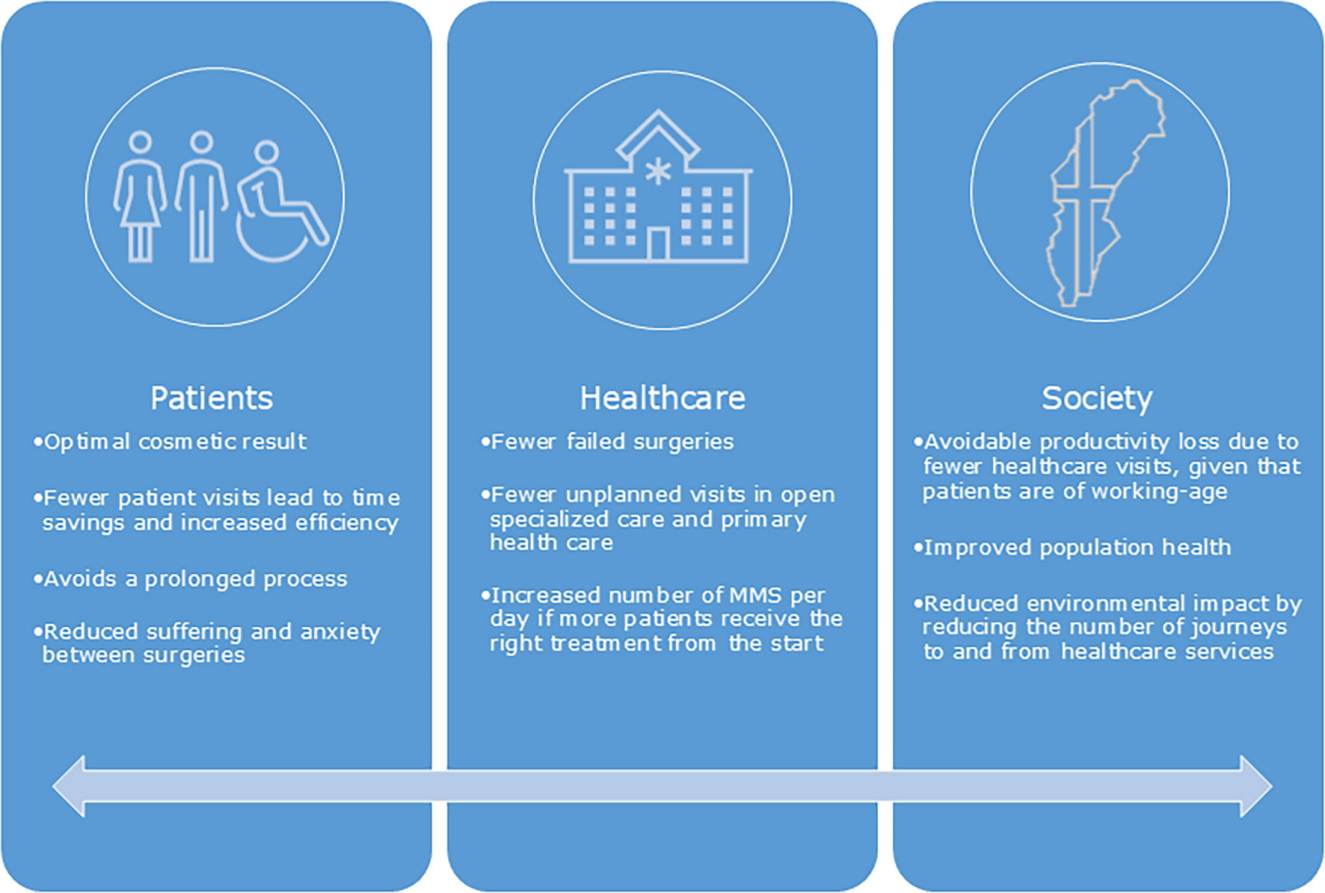
Potential benefits with increased MMS capacity for patients, healthcare and society.

Moreover, repeated interventions also increase indirect costs, including patient time, travel and productivity loss and other important factors such as cosmetic or functional impairment and psychological burden. This study focuses on healthcare costs and does also not include clinical outcomes such as recurrence or re-interventions. These aspects were not captured in this register-based analysis. Accounting for these factors would likely favour MMS as the more cost-favourable option from a health economic perspective (Fig. 2). Most prior studies have typically used quality-adjusted life years (QALYs) as the primary outcome measure (33), whereas we focused on actual healthcare costs and resource use. Incorporating factors that are not valued in monetary terms – such as reduced patient suffering, saved time for the patient, decreased anxiety and concern about recurrence and optimal cosmetic outcome – would more clearly demonstrate the overall value of MMS.

The underuse of MMS, despite its recognized safety and effectiveness, is a major problem. In Swedish clinical practice, MMS has traditionally been considered as a treatment of final option, primarily reserved for complex or recurrent cases. Clinicians often favour repeated SEs over MMS, reflecting limited awareness of its indications and benefits. This conservative approach, common across the Nordic countries, has contributed to limited access. The current waiting time of up to 12 months for MMS remains problematic. Such delays cannot reasonably be explained by limited resources but rather reflects suboptimal prioritization within the healthcare system.

Earlier use of MMS may reduce repeated procedures and improve resource efficiency. MMS achieves tumour clearance in a single procedure and is most efficient when used as primary treatment. Its effectiveness is reduced in recurrent cases, which often require >4 stages (34) and have higher recurrence risk (17, 20, 21, 35). Each additional procedure increases both clinical risk and resource use.

A previous study from our department showed an increase in primary MMS use and a reduction in surgical stages over time, along with smaller defect sizes and higher rates of primary closure (8). These findings support improved efficiency with earlier use of MMS.

Despite being considered the gold standard for high-risk facial BCC, MMS remains underutilized in Sweden. Further studies integrating both clinical outcomes and societal costs are needed to fully evaluate its value in routine practice.

### Strengths and limitations

The main strength of this study is the use of real-world healthcare resource utilization and cost data from the Vega regional healthcare registry. A further strength is the inclusion of all consecutive cases in cohort 1 from the local Mohs registry, ensuring complete capture of treated patients at our center.

Limitations include potential misclassification related to ICD-10 coding and the exclusion of indirect costs. In addition, the single-centre study design may limit generalizability to other settings, and not all tumours in cohort 2 may have been pathologically verified.

### Conclusion

This study suggests that MMS may be a cost-efficient treatment option for high-risk facial BCCs when used in accordance with Swedish treatment guidelines. Although MMS was associated with higher initial costs compared with SE, the long-term cost trajectory appeared more stable. Patients undergoing SE followed by MMS had the highest cumulative costs, indicating that treatment failure may contribute to increased healthcare expenditure over time. Differences in costs were not explained by baseline characteristics in adjusted analyses. These findings support the importance of considering long-term resource use when evaluating treatment strategies for high-risk facial BCC.

## Data Availability

The study used pseudonymized individual-level health data from the Swedish regional Vega database, approved by the ethics board and data controller. Due to legal and GDPR-related restrictions, these sensitive data cannot be publicly shared. The data that support the findings of this study are available from the corresponding author upon reasonable request.
